# Muscle Shear Moduli Changes and Frequency of Alternate Muscle Activity of Plantar Flexor Synergists Induced by Prolonged Low-Level Contraction

**DOI:** 10.3389/fphys.2017.00708

**Published:** 2017-09-20

**Authors:** Ryota Akagi, Takahito Fukui, Masato Kubota, Masashi Nakamura, Ryoichi Ema

**Affiliations:** ^1^College of Systems Engineering and Science, Shibaura Institute of Technology Saitama, Japan; ^2^Graduate School of Engineering and Science, Shibaura Institute of Technology Saitama, Japan; ^3^Japan Society for the Promotion of Science (JSPS) Tokyo, Japan

**Keywords:** muscle fatigue, joint torque, shear wave ultrasound elastography, evoked torque, electromyography

## Abstract

During prolonged low-level contractions, synergist muscles are activated in an alternating pattern of activity and silence called as alternate muscle activity. Resting muscle stiffness is considered to increase due to muscle fatigue. Thus, we investigated whether the difference in the extent of fatigue of each plantar flexor synergist corresponded to the difference in the frequency of alternate muscle activity between the synergists using muscle shear modulus as an index of muscle stiffness. Nineteen young men voluntarily participated in this study. The shear moduli of the resting medial and lateral gastrocnemius muscles (MG and LG) and soleus muscle (SOL) were measured using shear wave ultrasound elastography before and after a 1-h sustained contraction at 10% peak torque during maximal voluntary contraction of isometric plantar flexion. One subject did not accomplish the task and the alternate muscle activity for MG was not found in 2 subjects; therefore, data for 16 subjects were used for further analyses. The magnitude of muscle activation during the fatiguing task was similar in MG and SOL. The percent change in shear modulus before and after the fatiguing task (MG: 16.7 ± 12.0%, SOL: −4.1 ± 13.9%; mean ± standard deviation) and the alternate muscle activity during the fatiguing task (MG: 33 [20–51] times, SOL: 30 [17–36] times; median [25th–75th percentile]) were significantly higher in MG than in SOL. The contraction-induced change in shear modulus (7.4 ± 20.3%) and the alternate muscle activity (37 [20–45] times) of LG with the lowest magnitude of muscle activation during the fatiguing task among the plantar flexors were not significantly different from those of the other muscles. These results suggest that the degree of increase in muscle shear modulus induced by prolonged contraction corresponds to the frequency of alternate muscle activity between MG and SOL during prolonged contraction. Thus, it is likely that, compared with SOL, the alternate muscle activity of MG occurs more frequently during prolonged contraction due to the greater increase in fatigue of MG induced by the progression of a fatiguing task.

## Introduction

Human joint movement is produced by multiple muscles that act as synergists. During prolonged low-level contractions, the synergist muscles are activated in an alternating pattern of activity and silence (Hellsing and Lindström, 1983; Sjøgaard et al., 1986; Tamaki et al., 1998, 2011; Semmler et al., 1999, 2000; Kouzaki et al., 2002; Akima et al., 2012), referred to as alternate muscle activity (Kouzaki et al., 2002). The system of alternate activity among the synergists provides muscles with time to recover from the fatigue that develops during prolonged contraction (Tamaki et al., 2011). During prolonged (more than 1 h) low-level contraction, the alternate muscle activity was found to occur more frequently in the latter half than in the former half of the prolonged contraction to maintain the target level of joint torque in previous studies (Tamaki et al., 1998; Kouzaki et al., 2002; Akima et al., 2012). This finding is considered to be affected by the muscle fatigue (Tamaki et al., 1998) because it increases as the fatiguing task progresses. In addition, some previous studies (Enoka and Stuart, 1992; Gandevia, 2001; Kouzaki and Shinohara, 2006) have suggested that the alternate muscle activity between the synergist muscles is effective for minimizing or attenuating muscle fatigue. Therefore, there is a possibility that the difference in the fatigue of each synergist corresponds to the difference in the frequency of alternate muscle activity among the synergists.

Resting muscles become stiffer under several conditions, including those involving cramps and damage (Fischer, 1987; Murayama et al., 2000). Similarly, muscle fatigue has been considered as one of possible causes for the increment of muscle stiffness (Morisada et al., 2006; Descarreaux et al., 2010). It is known that a resting tension develops when muscles fail to fully relax during fatigue (Gong et al., 2000, 2003). Given that muscle tension increases muscle stiffness (Dresner et al., 2001), it is reasonable to assume that resting muscle stiffness increases due to muscle fatigue. Recently, resting muscle shear modulus was calculated by shear wave propagation speed within a muscle, which is simply and non-invasively determined by shear wave ultrasound elastography, and has been used as an index of muscle stiffness (Eby et al., 2015; Dieterich et al., 2017). The combination of this new technique and traditional methods, such as surface electromyography (EMG) and evoked torque/force, can be useful to examine the connection between the extent of fatigue of each synergist and the frequencies of alternate muscle activity among the muscles.

In the plantar flexors, alternate muscle activity occurs frequently during prolonged low-level contraction (more than 1 h; Tamaki et al., 1998, 2011; Kishibuchi and Kouzaki, 2013). It has been demonstrated that the medial gastrocnemius and soleus muscles (MG and SOL) play a key role in producing target plantar flexion torque during prolonged contraction compared to the lateral gastrocnemius muscle (LG) (Tamaki et al., 1998, 2011); possibly producing the greater fatigue of MG and SOL compared with LG after the prolonged contraction. To prevent the fatigue of MG and SOL, the alternate muscle activity of MG and SOL may occur more frequently compared with that of LG during the prolonged contraction. In this study, we determined the shear moduli of the plantar flexor synergists before and after 1-h sustained low-level contraction using shear wave ultrasound elastography and tested the following two hypotheses: (1) The contraction-induced increases in the shear moduli of MG and SOL are more prominent than that of LG and (2) of MG and SOL, the degree of increase in muscle shear modulus induced by prolonged contraction is larger in the muscle in which the alternate muscle activity occurs more frequently than in the other muscle.

## Materials and methods

### Subjects

After providing written informed consent, 19 healthy young men voluntarily participated in this study [age: 22 ± 1 year, height: 170.7 ± 4.5 cm, body mass: 62.6 ± 7.3 kg; mean ± standard deviation (*SD*)]. We confirmed existence or non-existence of the subjects' cardiovascular diseases and physical activity levels orally before starting the experiment. They were free of cardiovascular diseases. Also, they were sedentary and did not take any exercise. All measurements were performed with the subjects' right legs. This study was approved by the Ethics Committee of the Shibaura Institute of Technology and conducted according to the Declaration of Helsinki.

### Experimental procedures

**Figure 1:**
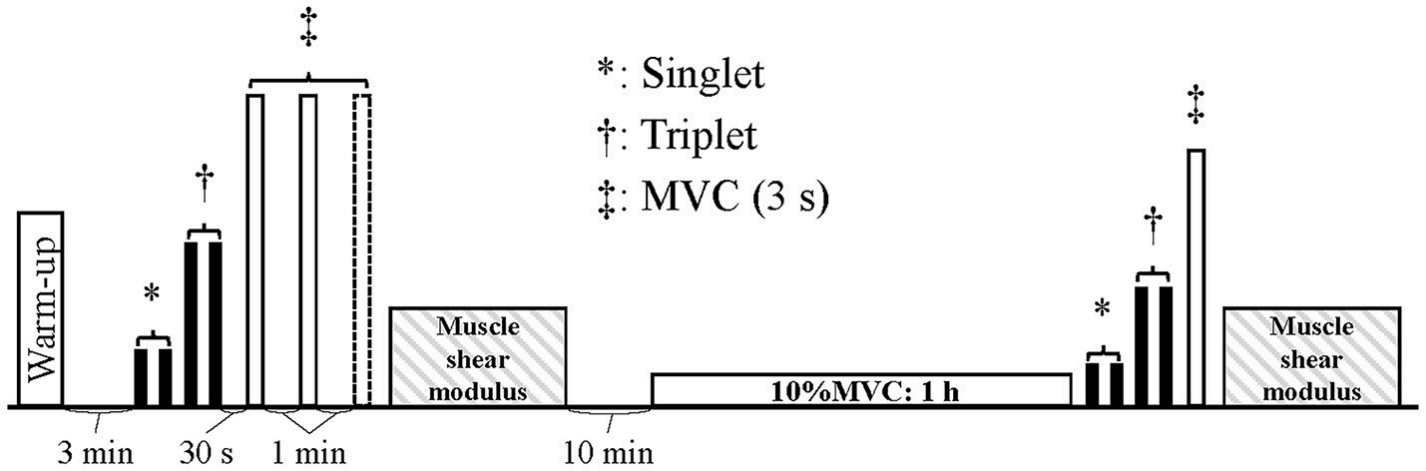
Experimental procedures. MVC, maximal voluntary contraction of isometric plantar flexion.

Figure 1 shows the experimental procedures. After determining the sites of surface EMG signals and tibial nerve stimulation, subjects performed several submaximal plantar flexion contractions as a warm-up and then rested for 3 min. Subsequently, the measurements of resting evoked singlet and triplet responses were performed every 10 s. After a 30-s rest period, the subjects performed maximal voluntary contraction (MVC) of isometric plantar flexion for 3 s two or three times. The measurements of the shear moduli of resting MG, LG, and SOL were performed after the MVC task. The subjects took a 10-min rest and started low-level (10%MVC) sustained contraction for 1 h as the fatiguing task. Immediately after finishing this task, the measurements of resting evoked singlet and triplet responses and the MVC task was repeated as described above. Last, the shear moduli of each muscle were measured.

### Shear modulus measurement

**Figure 2:**
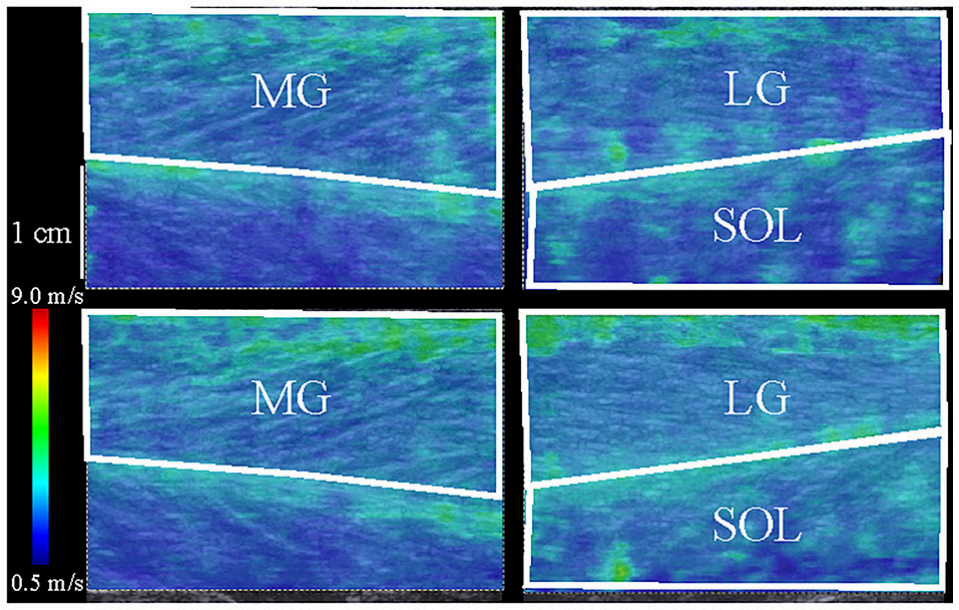
Typical elastographic images of the medial and lateral gastrocnemius muscles (MG and LG) and the soleus muscle (SOL) before (upper) and after (lower) the fatiguing task. Shear wave ultrasound elastography generated color-coded images with a scale from blue (soft) to red (hard) depending on the magnitude of shear wave propagation speed.

Both before and after performing the fatiguing task, the shear wave propagation speed within the resting MG, LG, and SOL was measured three times (Figure 2). The measurement sites of shear wave propagation speed within each muscle were determined as follows. For MG, at the proximal 40% of the lower leg length from the popliteal crease to lateral malleolus, the boundaries between MG and LG or the tibia were determined using ultrasonography. The elastographic image including MG was obtained at 40% of the girth from the boundary between MG and the tibia to that between MG and LG medially. For LG and SOL, the corresponding boundaries at the proximal 30% of the lower leg length were similarly determined. The elastographic image including both LG and SOL was obtained at 20% of the girth from the boundary between MG and the tibia to that between MG and LG laterally. At each measurement site, a 45-mm electronic linear array probe (9L4 Transducer, 4–9 MHz, Siemens Medical Solutions, USA) attached to a B-mode ultrasound apparatus (ACUSON S2000, Siemens Medical Solutions, USA) was longitudinally placed and its direction was adjusted to match the orientation of muscle fascicle before the first measurement of each shear wave propagation speed. Then, the measurement site was marked with a pen. Special care was taken to match the measurement site each time. The electronic linear array probe was placed at the measurement site with water-soluble transmission gel and without depression of the tissues. Three obtained elastographic images of sufficient quality were copied to a personal computer. The shear wave propagation speeds within MG, LG, and SOL were determined using image analysis software (MSI Analyzer version 2.0Aql, Institute of Rehabilitation Science, Tokuyukai Medical Corporation, Japan). A quadrangular region of interest (ROI) was set on the muscle to be as large as possible within the color-coded area of the elastographic image, and the mean value of the shear wave propagation speed within the ROI was automatically obtained at 0.01 m/s. The analyses of each image were conducted once. The mean values of the three measurements were used for further analyses. The coefficients of variance and intraclass correlation coefficients type 1, 3 for these measurements were 2.0 ± 1.1% and 0.955 (*P* < 0.001) for MG, 2.4 ± 2.1% and 0.966 (*P* < 0.001) for LG and 3.7 ± 1.9% and 0.912 (*P* < 0.001) for SOL.

The shear modulus of a muscle is calculated as the product of muscle density and shear wave velocity squared (Nordez and Hug, 2010; Akagi et al., 2015). In this study, the muscle density was assumed to be 1,084 kg/m^3^, which was the mean of the two values (1,112 and 1,055 kg/m^3^) obtained from different methods in a previous study (Ward and Lieber, 2005).

### EMG and joint torque measurements

Surface EMG signals were acquired from MG, LG, and SOL. The muscle belly and fascicle longitudinal directions were confirmed using a B-mode ultrasound apparatus (ACUSON S2000, Siemens Medical Solutions, USA) with the 45-mm electronic linear array probe (9L4 Transducer, 4–9 MHz, Siemens Medical Solutions, USA) perpendicular to the skin for identifying the fascicles. After skin shaving, rubbing with sandpaper and cleaning with alcohol, bipolar Ag/AgCl surface electrodes (F-150M, size adjustment by cutting to 10 × 20 mm, Nihon Koden, Tokyo, Japan; 20 mm inter-electrode distance) with high-pass filtering at 5 Hz using a bioamplifier system (MEG-6108, Nihon Koden, Tokyo, Japan) were placed at the proximal 40% (MG) and 30% (LG) of the lower leg length. The electrodes of MG and LG were set at 1 cm laterally from each site where the ultrasound probe was placed to determine the shear wave propagation speed within MG and LG before and after the fatiguing task. For SOL, electrode placement was ~5 cm distal from the proximal 40% of the lower leg length, but there were inter-individual variations due to the variability of the superficial region of SOL.

Muscle fatigue is generally defined as any exercise-induced reduction in the ability of a muscle to generate force or power (Gandevia, 2001). In this study, MVC torque (TQ_MVC_) and evoked peak triplet torque (TQ_TRI_) were determined because it is considered that the exercise-induced decrease in TQ_MVC_ depends on both peripheral and central factors (Fernandez-del-Olmo et al., 2013) whereas that in TQ_TRI_ reflects peripheral fatigue (Miyamoto et al., 2011).

To obtain TQ_TRI_ and the peak-to-peak compound muscle action potential amplitude (Mmax), the bioamplifier system (MEG-6108, Nihon Kohden, Japan) and a constant-current variable voltage stimulator (DS7A, Digitimer Ltd., UK) with a controller (SEN-3401, Nihon Kohden, Japan) were used. The tibial nerve was stimulated percutaneously with rectangular pulses of 1 ms in duration in the popliteal fossa with a cathode (2 × 2 cm). An anode (4 × 5 cm) was placed over the ventral aspect of the thigh, just proximal to the patella.

Before and after the fatiguing task, Mmax, TQ_TRI_, and TQ_MVC_ were measured. Subjects sat on a seat of a dynamometer (CON-TREX MJ, Physiomed, Germany) with the hip at 80° of flexion, the knee fully extended and the ankle at 20° of plantar flexion. This ankle joint angle was set based on a previous finding that the appropriate ankle position for detecting frequent alternations of activity in plantar flexor synergists was 20° of plantar flexion during static contractions at 10%MVC (Tamaki et al., 2011). The subject's pelvis, torso and ankle were secured on the reclining seat and dynamometer with non-elastic straps and/or seat belt. Care was taken to adjust centers of the rotation of the ankle joint and the dynamometer. Firstly, stimulus intensity was increased until a plateau in the twitch torque and Mmax were reached at 20° of plantar flexion and supramaximal stimulus intensity was set (66 ± 25 mA; mean ± *SD*). Then, two Mmax and two TQ_TRI_ (100 Hz) were obtained every 10 s, and averaged across the two contractions, respectively. Thereafter, the peak torque during MVC was measured two or three times with a 1 min interval. If the difference between the first two values of peak torque was >10% of the higher one, the peak torque was measured one more time. Before the fatiguing task, the highest value of two or three peak torque measurements was adopted as TQ_MVC_. After the fatiguing task, TQ_MVC_ measurement was performed only once. The EMG and joint torque signals were recorded at a sampling frequency of 2000 Hz and stored in a personal computer using LabChart software (v8.1.5, ADInstruments, Australia) after A/D conversion (PowerLab16/35, ADInstruments, Australia).

During the fatiguing task, the torque data were displayed as waveforms on a monitor of a personal computer using LabChart software in real time. The horizontal target line (i.e., 10%MVC) was also displayed on the monitor using the software, and the monitor was placed in front of the subjects to provide visual feedback. As described above, the subject's pelvis, torso and ankle were secured on the reclining seat and dynamometer with non-elastic straps and/or seat belt during the fatiguing task, but the fixation of the subject's ankle was unfixed slightly to avoid the numbness in the right leg or foot. The subjects tried to match their torque level and the target line, and were instructed not to alter joint angles during the fatiguing task. The ankle joint angle was determined with an electronic goniometer (SG110/A, Biometrics, UK).

### EMG data analysis

EMG signals during the MVC tasks were full-wave rectified and averaged 0.5 s in the period around the peak torque to calculate the average EMG signals (AEMG) in each MVC task. Each AEMG in the selected MVC tasks before and after the fatiguing task was used. Moreover, the AEMG was normalized by Mmax in each muscle both before and after the fatiguing task to minimize peripheral contamination and thus to assess the level of central motor output (Place et al., 2010).

**Figure 3:**
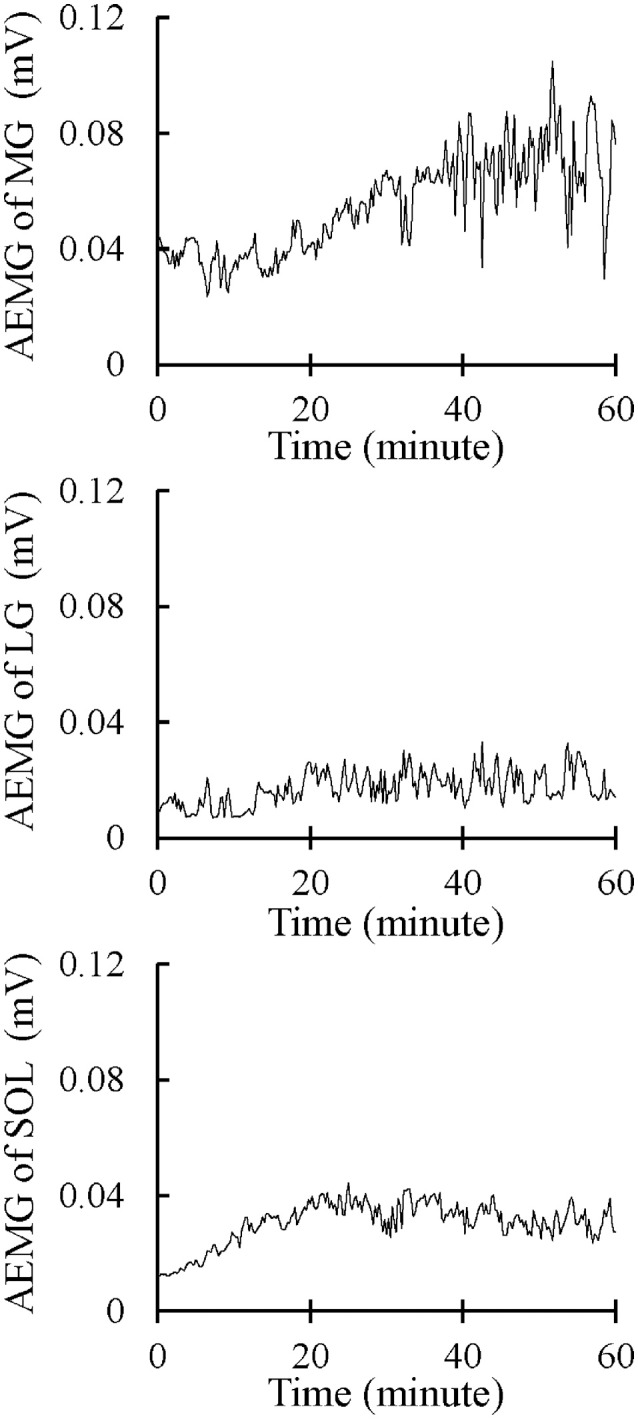
Typical images of the 15-s averaged electromyography signals (AEMG) of the medial and lateral gastrocnemius muscles (MG and LG) and the soleus muscle (SOL) during the 1-h fatiguing task.

**Figure 4:**
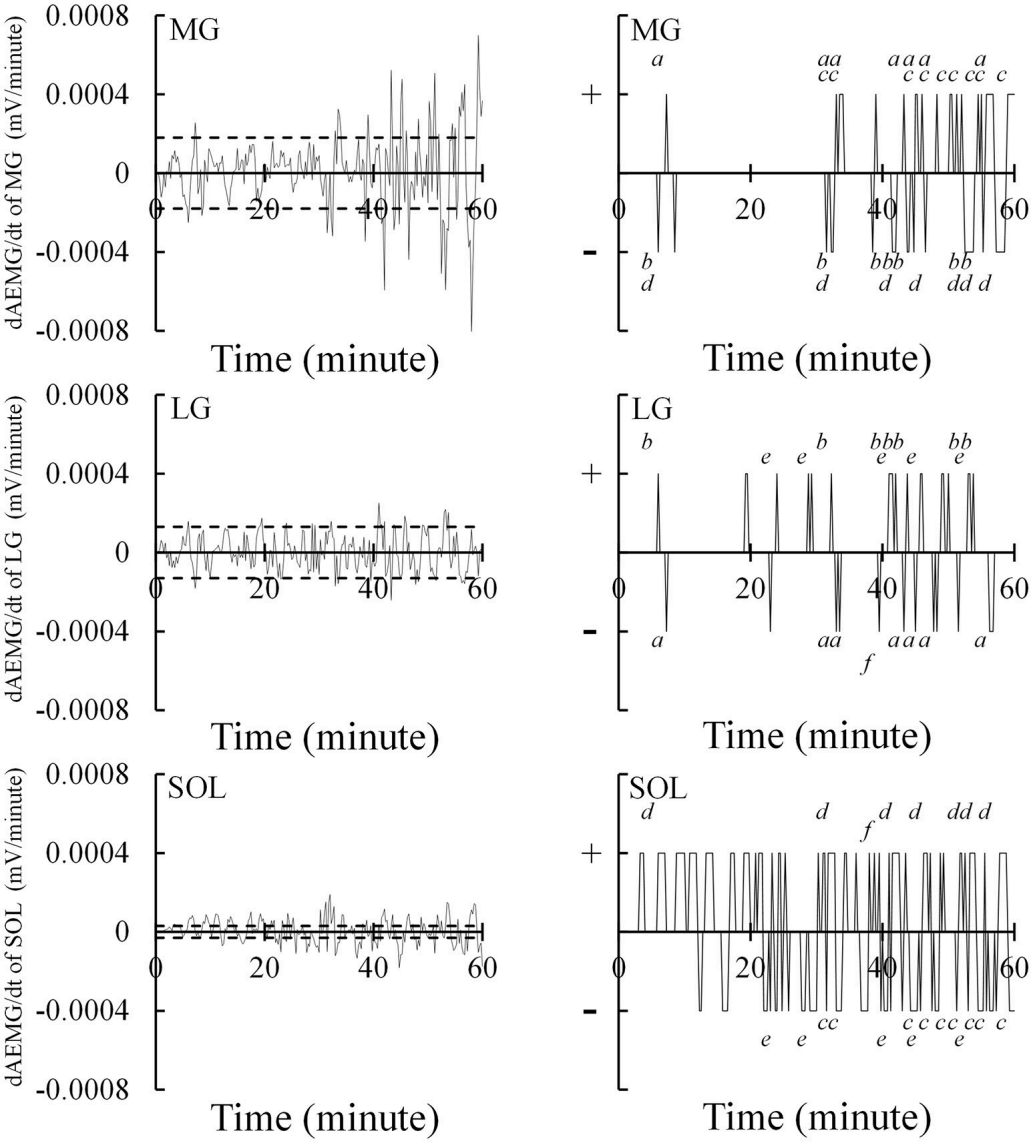
Measuring alternate muscle activity during the 1-h fatiguing task. The 15-s averaged electromyography signals of the medial and lateral gastrocnemius muscles (MG and LG) and the soleus muscle (SOL) were smoothed by five-point moving average and differentiated (dAEMG/dt). The left figures show the time-course changes in dAEMG/dt of each muscle. Eight sample points of dAEMG/dt immediately after the onset of exercise were extracted and 3 standard deviations (3 *SD*s) of dAEMG/dt during this period were determined as the normal fluctuation. The dashed line indicates the ±3 *SD*s. The criterion for an outlier was defined as dAEMG/dt throughout the fatiguing task that exceeded the dashed lines. As shown on the right, the extracted outliers were classified into positive (+) and negative (−) outliers per muscle. The alternate muscle activity of LG → MG, MG → LG, SOL → MG, MG → SOL, SOL → LG, and LG → SOL are indicated using *a, b, c, d, e*, and *f*, respectively.

Alternate muscle activity was observed through EMG activity between the plantar flexor synergists during the fatiguing task. The alternate muscle activity was defined and measured according to previously established methods (Kouzaki et al., 2002; Kouzaki and Shinohara, 2006). The EMG signals were full-wave rectified and averaged over 15 s to yield the AEMG every 15 s (Figure 3). The calculated AEMG of each muscle was smoothed by a five-point moving average and differentiated (dAEMG/dt). Eight sample points of dAEMG/dt immediately after the onset of exercise were extracted and 3 *SD*s of dAEMG/dt during this period were determined as a normal fluctuation. The criterion for an outlier was defined as dAEMG/dt throughout the fatiguing task that exceeds ±3 *SD*s, including both upper and lower limits as a normal fluctuation (Figure 4). The extracted outliers were classified into positive (+) and negative (−) outliers per muscle. The alternate muscle activity between the plantar flexor synergists was defined as the case in which the positive and the negative outliers were simultaneously observed between the muscles (Figure 4). The overlap in time for extraction of alternate muscle activity was accepted for a 15-s period. The alternate muscle activity between MG and LG (1), MG and SOL (2), and LG and SOL (3) was counted. In Figure 4, for example, (1) = numbers of *a* + *b* = 14, (2) = numbers of *c* + *d* = 16, and (3) = numbers of *e* + *f* = 6. In addition, the frequency of alternate muscle activity (N_0−60_) for each muscle was calculated as follows: MG = (1) + (2), LG = (1) + (3), SOL = (2) + (3). These frequencies were also counted every 30 min (N_0−30_ and N_30−60_). In each muscle, the AEMG every 15 s during the fatiguing task was normalized using the AEMG in the selected MVC task before the fatiguing task, and the mean value during the fatiguing task was calculated as %AEMG_0−60_.

### Statistical analyses

Of the 19 subjects, 1 was unable to complete the fatiguing task and alternate muscle activity for MG was not found in 2 participants because of the great variability in the 8 sample points of dAEMG/dt immediately after the onset of exercise. Consequently, the data for 16 subjects were used for further analyses.

A two-way analysis of variance (ANOVA) with two within-group factors (time [before and after the fatiguing task] and muscle [MG, LG, and SOL]) was used to evaluate changes induced by the fatiguing task in muscle shear moduli, Mmax, and AEMG during the MVC task normalized by Mmax. When a significant interaction was detected, an additional ANOVA with the Bonferroni multiple-comparison test was performed. The percentage changes in the shear moduli and normalized AEMG during the MVC task of each muscle from before to after the fatiguing task were also calculated and their differences were examined using a one-way ANOVA followed by the Bonferroni multiple-comparison test. Differences in TQ_MVC_ and TQ_TRI_ before and after the fatiguing task were examined using a paired *t*-test.

The one-way ANOVA followed by Bonferroni multiple-comparison test was used to investigate differences in %AEMG_0−60_ among MG, LG, and SOL. For the data of N_0−60_, N_0−30_, and N_30−60_ of each muscle, normality was tested using the Shapiro–Wilk test. As a result, some of N_0−60_, N_0−30_, and N_30−60_ of each muscle were not normally distributed, and therefore, differences in N_0−60_, N_0−30_, and N_30−60_ among MG, LG, and SOL were tested using the Friedman test followed by the Wilcoxon *post-hoc* test. In addition, relationships of N_0−60_, N_0−30_, or N_30−60_ and the percentage change in the muscle shear modulus were tested in each muscle using Spearman's rank correlation coefficients.

To test the effect of the individual variability of joint torque level during the fatiguing task on the fatigue-induced decline in joint torque, the data of joint torque during the fatiguing task were firstly normalized by TQ_MVC_ as the joint torque level during the fatiguing task. Then, the percentage changes in TQ_MVC_ and TQ_TRI_ from before to after the fatiguing task (%ΔTQ_MVC_ and %ΔTQ_TRI_) were computed, and Pearson's product-moment correlation coefficients were calculated between the joint torque level and %ΔTQ_MVC_ or %ΔTQ_TRI_.

Parametric data are presented as means ± *SD*s. Statistical significance was set at *P* < 0.05. Statistical analyses were performed using statistical analysis software (SPSS 24.0, IBM, USA).

## Results

### Shear moduli and joint torque

**Figure 5:**
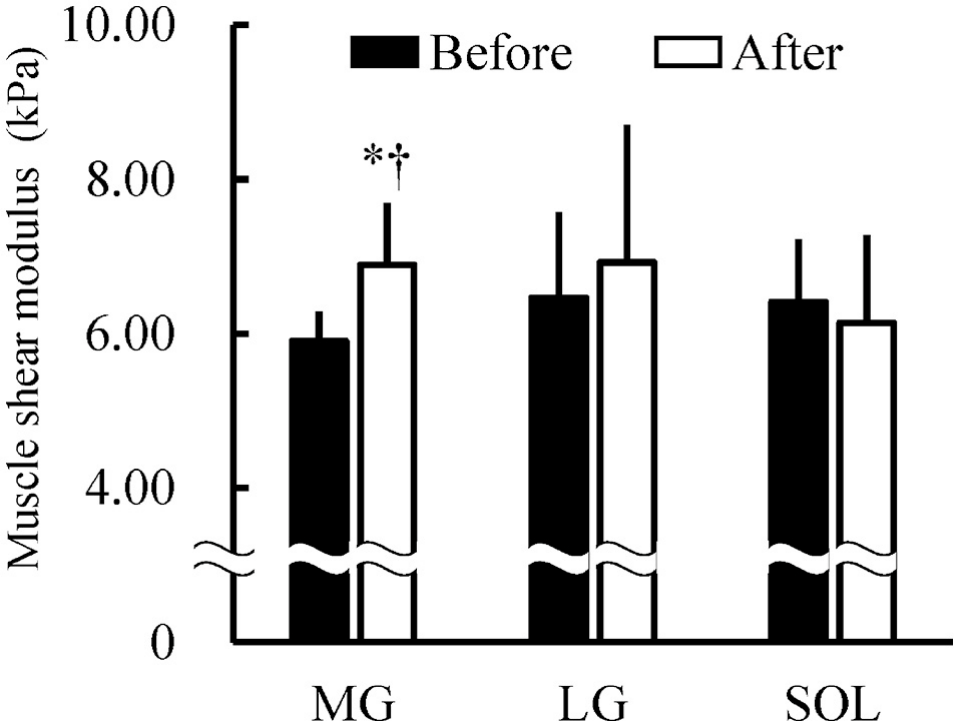
Shear moduli of the medial and lateral gastrocnemius muscles (MG and LG) and the soleus muscle (SOL) before and after the 1-h fatiguing task (*n* = 16). A two-way analysis of variance revealed that there were a significant main effect of muscle and a significant time × muscle interaction. ^*^Indicates a significant difference before and after the fatiguing task (*P* < 0.05). ^†^Indicates a significant difference between MG and SOL (*P* < 0.05).

**Figure 6:**
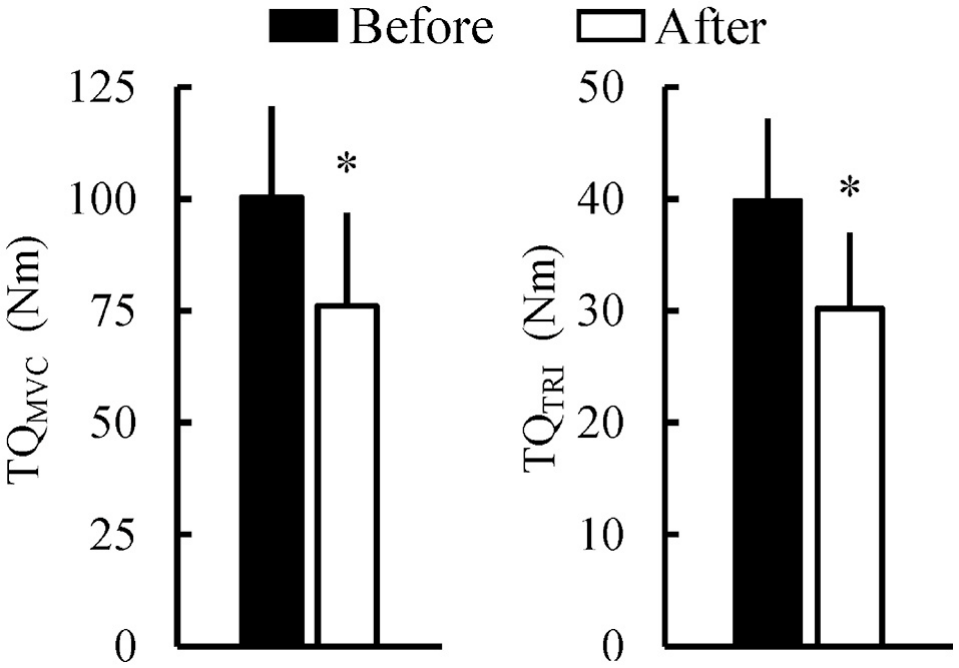
Peak joint torque during maximal voluntary contraction of isometric plantar flexion (TQ_MVC_) and evoked peak triplet torque (TQ_TRI_) before and after the 1-h fatiguing task (*n* = 16). ^*^Indicates the significant difference before and after the fatiguing task (*P* < 0.05).

Figure 5 shows the muscle shear moduli before and after the fatiguing task. A significant time × muscle interaction (*P* = 0.001) was found without significant main effects of muscle (*P* = 0.057) or time (*P* = 0.277). The shear modulus of MG significantly increased after the fatiguing task (*P* < 0.001), but there were no significant differences in the shear modulus before and after the fatiguing task in LG (*P* = 0.193) and SOL (*P* = 0.258). In addition, the shear modulus of MG was significantly higher than that of SOL only after the fatiguing task (*P* = 0.049). Correspondingly, for the percentage change in the muscle shear moduli from before to after the fatiguing task, a main effect of muscle was significant (*P* = 0.028), and the percentage change in MG (16.7 ± 12.0%) was significantly higher than that in SOL (−4.1 ± 13.9%; *P* < 0.001), but not than that in LG (7.4 ± 20.3%; *P* = 0.306). TQ_MVC_ and TQ_TRI_ were significantly decreased after the fatiguing task (both *P* < 0.001; Figure 6).

### EMG

**Figure 7:**
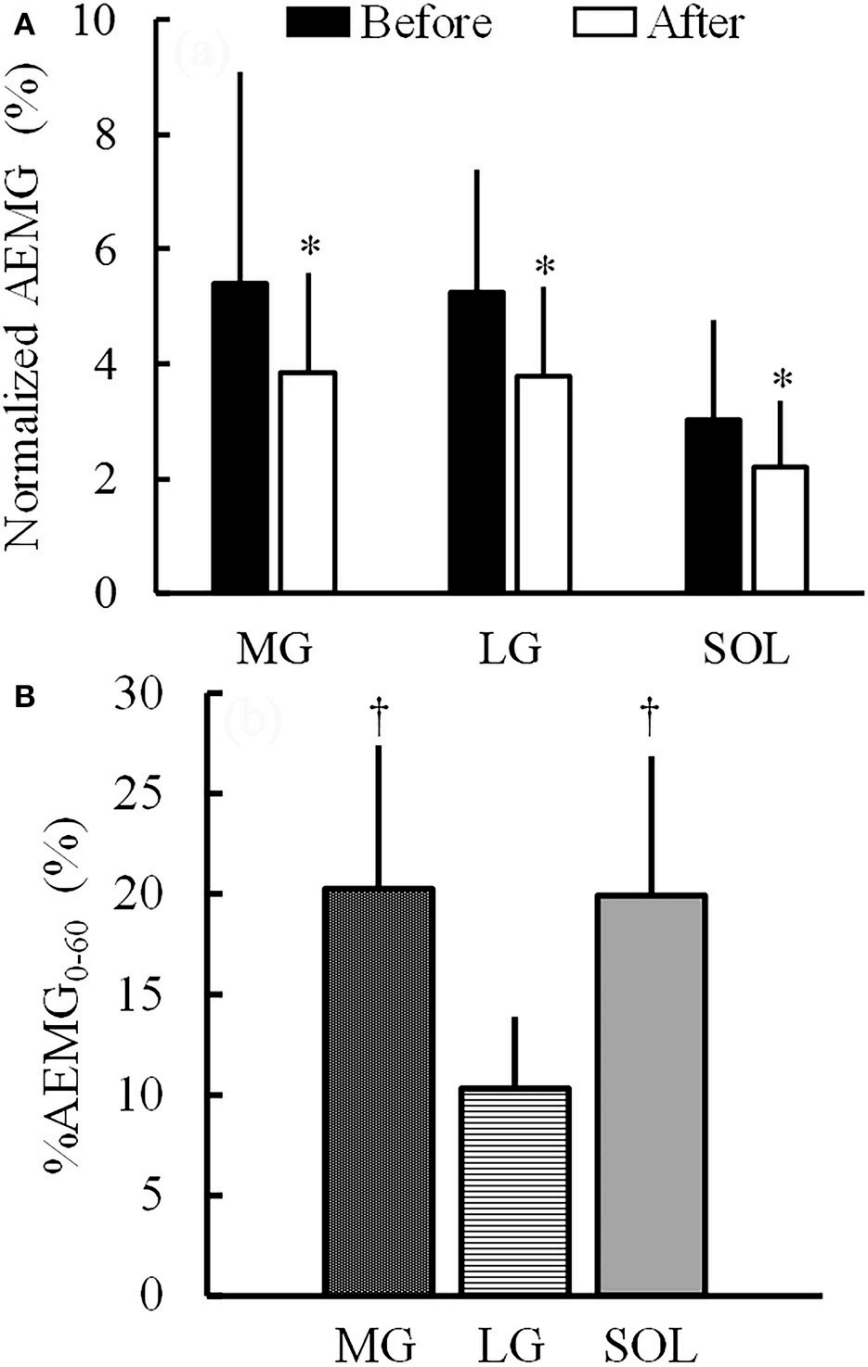
Average electromyographic signal (AEMG) during the maximal voluntary contraction normalized by the peak-to-peak compound muscle action potential amplitude **(A)** and AEMG during the 1-h fatiguing task normalized by AEMG in the maximal voluntary contraction task before the 1-h fatiguing task (%AEMG_0−60_) **(B)** (*n* = 16). MG, the medial gastrocnemius muscle; LG, the lateral gastrocnemius muscle; SOL, the soleus muscle. ^*^Indicates the significant main effect of time (before and after the 1-h fatiguing task) (*P* < 0.05) without a significant time × muscle interaction. Regarding %AEMG_0−60_, there was a significant main effect of muscle. ^†^Indicates the significant difference between MG or SOL and LG (*P* < 0.05).

For Mmax, there was neither a significant main effect of time (*P* = 0.823) nor a significant time × muscle interaction (*P* = 0.825). In respect to AEMG during the MVC task normalized by Mmax, there was a significant main effect of time (*P* = 0.009) without a significant time × muscle interaction (*P* = 0.280) (Figure 7A). There was no significant main effect of muscle on the percent changes in normalized AEMG during the MVC task (MG: −20.6 ± 29.9%; LG: −23.8 ± 20.2%; SOL: −21.1 ± 26.6%; *P* = 0.880). Regarding %AEMG_0−60_, a significant main effect of muscle was found (*P* < 0.001), and %AEMG_0−60_ of LG (10.3 ± 3.6%) was significantly lower than those of MG (20.3 ± 7.1%; *P* < 0.001) and SOL (19.9 ± 6.9%; *P* = 0.001; Figure 7B).

### Frequency of alternate muscle activity

**Figure 8:**
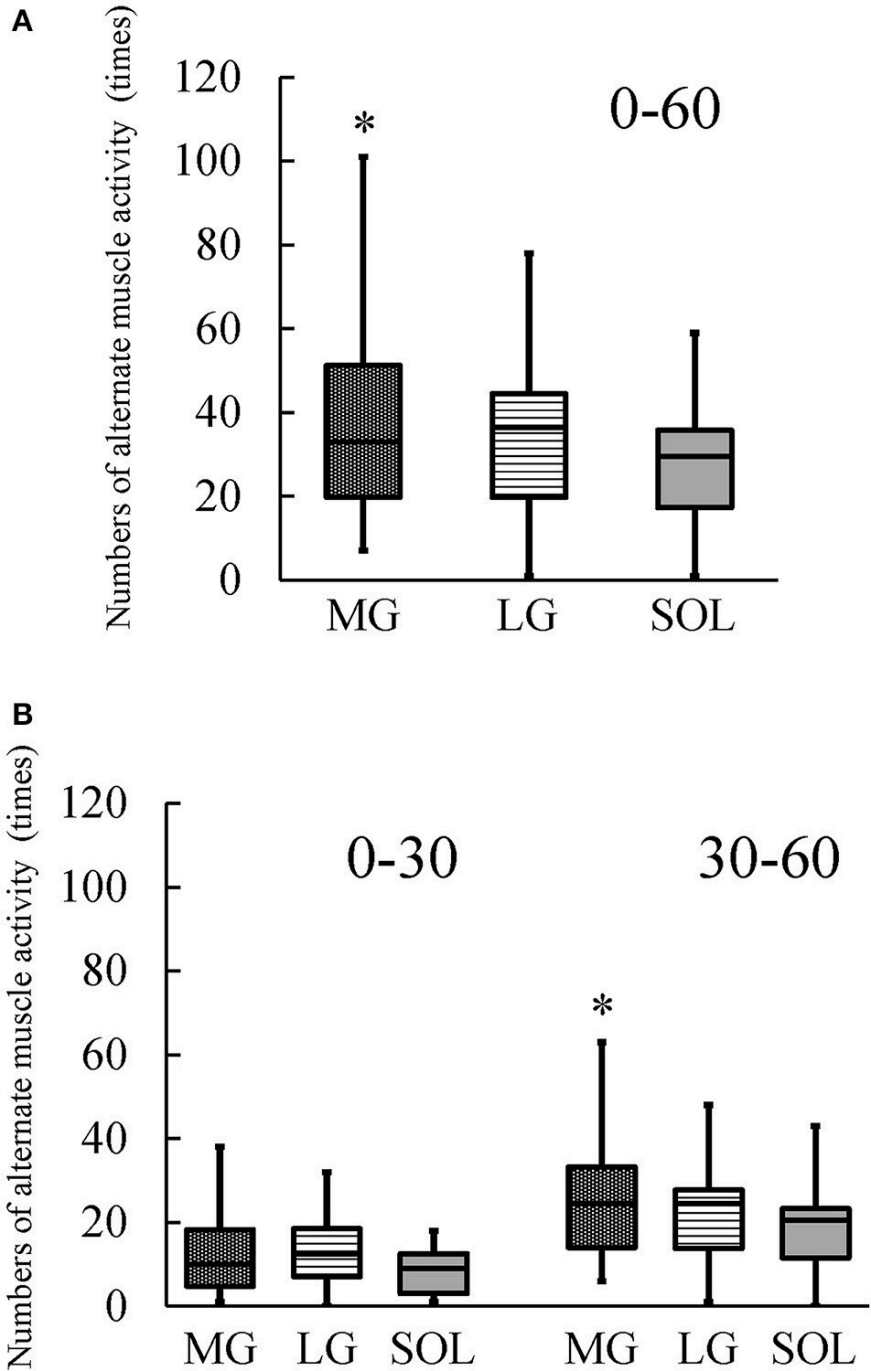
Numbers of alternate muscle activity of the medial and lateral gastrocnemius muscles (MG and LG) and the soleus muscle (SOL) during the 1-h fatiguing task (0–60) **(A)** and every 30 min (0–30 and 30–60) (*n* = 16) **(B)**. A boxplot shows median, 25th and 75th percentiles, and minimum and maximum values. The Friedman test revealed that there was a significant main effect of muscle. ^*^Indicates the significantly higher numbers of alternate muscle activity of MG than of SOL (*P* < 0.05).

For N_0−60_ and N_30−60_, a main effect of muscle was significant (N_0−60_: *P* = 0.022; N_30−60_: *P* = 0.006). Wilcoxon *post-hoc* test revealed that N_0−60_ and N_30−60_ were significantly higher in MG than in SOL (N_0−60_: *P* = 0.040; N_30−60_: *P* = 0.006; Figure 8). Regarding N_0−30_, a significant main effect of muscle was not found (*P* = 0.074; Figure 8). When examining relationships between the frequency of alternate muscle activity and the percentage change in the muscle shear modulus, none of the correlations were significant (MG: *r*_s_ = 0.284, *P* = 0.286 [N_0−60_]; *r*_s_ = 0.225, *P* = 0.402 [N_0−30_]; *r*_s_ = 0.066, *P* = 0.807 [N_30−60_]; LG: *r*_s_ = 0.397, *P* = 0.128 [N_0−60_]; *r*_s_ = 0.489, *P* = 0.054 [N_0−30_]; *r*_s_ = 0.440, *P* = 0.088 [N_30−60_]; SOL: *r*_s_ = 0.068, *P* = 0.803 [N_0−60_]; *r*_s_ = −0.040, *P* = 0.883 [N_0−30_]; *r*_s_ = −0.317, *P* = 0.231 [N_30−60_]).

### Variability in ankle joint angle and joint torque during the fatiguing task

The SD of change in ankle joint angle during the fatiguing task was 1.6 ± 1.8° (min: 0.2°, max: 7.3°). The mean values and SD of joint torque level during the fatiguing task were 9.5 ± 0.6% (min: 8.7%, max: 11.1%) and 1.1 ± 0.5% (min: 0.4%, max: 1.9%). There were no significant correlations between the joint torque level during the fatiguing task and %ΔTQ_MVC_ (*r* = −0.272, *P* = 0.309) or %ΔTQ_TRI_ (*r* = −0.391, *P* = 0.134).

## Discussion

After sustaining 10%MVC for 1 h, both TQ_MVC_ and TQ_TRI_ were significantly decreased (Figure 6). It is as that the extent of the prolonged contraction-induced decrease in TQ_TRI_ reflects peripheral fatigue (Miyamoto et al., 2011) whereas that in TQ_MVC_ depends on both peripheral and central factors (Fernandez-del-Olmo et al., 2013). Therefore, both peripheral and central fatigue are suggested to result in the decline in joint torque after the fatiguing task. N_0−60_ and N_30−60_ were significantly higher in MG than in SOL; however, there was no significant difference in N_0−30_ among the plantar flexors (Figure 8). These results suggest that the difference in the frequency of alternate muscle activity among the plantar flexors is prominent in the latter half of the prolonged contraction because muscle fatigue increases as the fatiguing task progresses. Based on these suggestions, we would like to refer to the fatigue-induced changes in the shear moduli of the plantar flexor synergist.

In the current study, the %AEMG_0−60_ was significantly higher in MG and SOL than in LG (Figure 7B), which is in agreement with previous findings (Tamaki et al., 1998, 2011). Considering the present results and the previous findings that the ratio of LG volume to total muscle volume of MG and SOL was <20% (Fukunaga et al., 1992), it is reasonable to suggest that MG and SOL contributed more greatly to sustaining 10%MVC during the fatiguing task compared with LG. The hypotheses of the current study were that (1) the increases in the shear moduli of MG and SOL are more prominent than that of LG after the prolonged contraction and (2) of MG and SOL, the alternate muscle activity occurs more frequently in the muscle with the larger degree of increase in muscle shear modulus induced by the prolonged contraction. In the present study, the significant increase in shear modulus after the fatiguing task was found only in MG (Figure 5) but the percent change in shear modulus of MG was not significantly higher than that of LG. Furthermore, there was no significant difference in the percent change in shear modulus between LG and SOL. Thus, the first hypothesis was rejected. Meanwhile, N_0−60_ and N_30−60_ were significantly higher in MG than in SOL (Figure 8) and the increase in shear modulus induced by the fatiguing task was found in MG but not in SOL (Figure 5). Additionally, the percent change in shear modulus of MG was significantly higher than that of SOL. These results indicate that the fatigue-induced increase in muscle shear modulus occurred in MG but not in SOL, supporting the second hypothesis.

To clarify the reasons for the rejection of the first hypothesis, we would like to discuss the results of MG and LG and those of LG and SOL separately. In respect to MG and LG, the significant increase in shear modulus after the fatiguing task was found in MG but not in LG (Figure 5). As described above, MG is suggested to contribute more greatly to sustaining 10%MVC during the fatiguing task compared with LG. Therefore, despite no significant differences in the frequency of alternate muscle activity between MG and LG (Figure 8), the difference in the contribution to sustaining 10%MVC between them appears to influence the current results of their shear moduli (Figure 5). Regarding LG and SOL, the contribution of LG to sustaining 10%MVC during the fatiguing task seemed to be lower than that of SOL whereas the fatigability is greater for LG than for SOL (Ochs et al., 1977; Sypert and Munson, 1981). Hence, fatigue of LG can be regulated by the lower contribution of LG to sustaining 10%MVC and the greater fatigability for LG compared with SOL, likely affecting the current results that there were no significant differences in the shear moduli before and after the fatiguing task (Figure 5) and in N_0−60_, N_0−30_, and N_30−60_ between LG and SOL (Figure 8).

As a reason for supporting the second hypothesis, the effect of the difference in fatigability between MG and SOL on the difference in frequency of alternate muscle activity during the prolonged low-level contraction between them is cited. Physical fluctuations of the muscle of ~8–12 Hz, which are termed physiological tremors and arise from Ia afferent activity and neural oscillations from central commands to motoneuron pool, have been observed in fatigued muscles (McAuley and Marsden, 2000). In accordance with a previous study (Kouzaki et al., 2004) investigating the physiological tremor of knee extension force fluctuations during 1-h knee extension at 2.5% of MVC, its change has been suggested to be caused by the unique muscle activity of the rectus femoris muscle (RF) during the alternate muscle activity due to the more fatigable RF than the other synergists. Another study (Kouzaki and Shinohara, 2006) has also pointed out that, for the alternate muscle activity among the knee extensors, fatigue-related feedback information is likely to be transmitted from the afferents of RF to α-motoneurons in each knee extensor muscle, most likely via interneurons, leading to the alternate muscle activity. Thus, the more fatigable RF compared with the other synergists is expected to be a key muscle for the alternate muscle activity among the knee extensors during prolonged contractions in low-level. In fact, the alternative muscle activity appears to occur more frequently in RF than in the other synergists during such contractions as shown previously (Kouzaki et al., 2002; Akima et al., 2012). In a previous study investigating the plantar flexors (Kishibuchi and Kouzaki, 2013), alternate muscle activity during a 2-h prolonged contraction in 10%MVC was associated with changes in physiological tremor of ankle angular acceleration when the AEMG of MG dramatically decreased with the increase in the AEMG of LG and/or SOL. In particular, the activity of MG, but not the activity of the other synergists, was accompanied by physiological tremor. Hence, Kishibuchi and Kouzaki (2013) have demonstrated that these findings would correspond to the findings of RF described above (Kouzaki et al., 2004), and that MG is a key muscle for the alternate muscle activity among the plantar flexors during the prolonged low-level contraction. MG has been suggested to have the greater fatigability compared with SOL based on the previous findings of their contraction-induced fatigue and/or fiber type composition (Ochs et al., 1977; Sypert and Munson, 1981). In addition, the higher N_0−60_ and N_30−60_ of MG than of SOL were found in the current study (Figure 8). Therefore, considering the similarity between MG and RF from the perspective of fatigability, it is possible that the increase in the muscle shear modulus after the fatiguing task was found in the more fatigable MG but not in the less fatigable SOL in the present study (Figure 5).

On the other hand, there were no significant correlations between N_0−60_, N_0−30_, or N_30−60_ and the percentage change in the muscle shear modulus in any plantar flexors. As described in the Section Introduction, the system of alternate activity among the synergists provides muscles with time to recover from the fatigue that develops during prolonged contraction (Tamaki et al., 2011). However, it is not clear whether the degree of recovery from the fatigue induced by the alternate muscle activity is consistent from person to person. If this is inconsistent among individuals, the relationships between the frequency of alternate muscle activity and the percentage change in the muscle shear modulus should be weakened. In addition, based on previous findings (Tamaki et al., 1998, 2011), Kishibuchi and Kouzaki (2013) indicated that alternate muscle activity of plantar flexors emerges without regularity, and that overlapped activities between the muscles are observed in the plantar flexors. Thus, the alternations of plantar flexor during prolonged contraction are considered to be complicated. This complexity may also weaken the aforementioned relationships. Taken together, the present results suggest the difficulty of comparing the effect of the frequency of alternate muscle activity on the muscle shear modulus among individuals. In other words, the second hypothesis is likely to be supported based on the results of group means, regardless of the results of the correlations between muscle shear modulus and the frequency of alternate muscle activity.

We discuss implications of the present findings in this part. During the 1-h fatiguing task, the magnitudes of muscle activations of MG and SOL were higher than that of LG. Based on this result and the previous findings that the ratio of LG volume to total muscle volume of MG and SOL was <20% (Fukunaga et al., 1992), MG and SOL can be considered to contribute more greatly to perform plantar flexion compared with LG as described in the earlier part. On the other hand, of MG and SOL, the task-induced increase in muscle fatigue evaluated by shear wave ultrasound elastography was found only in MG. In other words, the muscle fatigue of SOL during the task was likely to be attenuated by the alternate muscle activity. Thus, the difference in fatigability was prominent between MG and SOL during performing plantar flexion. Therefore, when conducting a fatigue-training of the plantar flexor synergists, for instance, the differences in the contributions to plantar flexion strength and the fatigue profiles of the three muscles could imply inter-muscle differences in the training modality. Furthermore, it has been reported that the gastrocnemius muscle is more susceptible to injury than SOL, with by far the majority of the strain injuries involving MG as opposed to LG (Koulouris et al., 2007). Hence, when performing exercises as sports or recreational activities which could result in the muscle fatigue of the plantar flexors, special care may be needed for the muscle fatigue of MG.

The present study had three limitations. First, there was variability in the ankle joint angles and joint torque level during the 1-h fatiguing task. Tamaki et al. (2011) found that there were no significant differences in the averaged EMG activities of MG, LG, and SOL during MVC between the ankle joint angles of 20° and 10° or 30° of plantar flexion. Because the ankle joint angle of 20° of plantar flexion was used in the current study, it is unlikely that the interpretation of the current study was greatly influenced by the changes in the ankle joint angle observed here (SD of change in ankle joint angle during the fatiguing task: 1.6 ± 1.8° [0.2–7.3°]). The current results suggest that the target level of joint torque was roughly maintained during the fatiguing task. Moreover, judging from the result that there were no significant correlations between the joint torque level during the fatiguing task and %ΔTQ_MVC_ or %ΔTQ_TRI_, the effect of the individual variability of joint torque level during the fatiguing task on the fatigue-induced decline in joint torque is likely to be small. These results indicate that the effects of variability in the ankle joint angles and joint torque level during the fatiguing task are ignorable in this study. Second, changes in muscle water content would affect the values of muscle shear moduli because the subject's posture was nearly unchanged through the experiment (i.e., for over 1 h). This fact may influence the differences in muscle shear moduli before and after the fatiguing task in each muscle. However, it is hard to think that there was a large difference in the change in muscle water content among the plantar flexors. In other words, it is very difficult to explain the reason why the shear modulus significantly increased after the fatiguing task only in MG from the standpoint of change in muscle water content. Therefore, the effect of the changes in muscle water content on the interpretation of the present results seems to be small. Third, changes in muscle blood volume was not considered in the present study. Kouzaki et al. (2003) investigated the relationship between local blood circulation and alternate muscle activity in RF and vastus lateralis muscle during 1-h knee extension at 2.5%MVC. In the previous study, local blood circulation was modulated by the alternate muscle activity of knee extensor synergists, and a negative correlation between the muscle activity and blood volume sequences was found in the more fatigable RF but not in vastus lateralis muscle. This phenomenon can also be observed among the plantar flexor synergists during the prolonged contraction. If so, the data of muscle shear modulus in the present study might be affected by the muscle blood volume. This effect needs to be investigated in the future to strengthen the present findings.

In summary, the magnitude of muscle activation during the 1-h fatiguing task was similar in MG and SOL. The muscle shear modulus increased after the 1-h fatiguing task only in MG and the percent change in shear modulus of MG from before to after the fatiguing task was higher than that of SOL. Furthermore, N_0−60_ and N_30−60_ were higher in MG than in SOL. The contraction-induced change in shear modulus and alternate muscle activity during the prolonged contraction of LG, which had the lowest magnitude of muscle activation during the fatiguing task among the plantar flexors were not significantly different from those of the other muscles. These results suggest the correspondence of the degree of increase in muscle shear modulus induced by prolonged contraction to the frequency of alternate muscle activity especially in the latter half of the prolonged contraction between MG and SOL. Thus, based on the results of muscle shear modulus, it can be concluded that, compared with SOL, the alternate muscle activity of MG occurs more frequently—especially in the latter half of the prolonged contraction due to the greater increase in fatigue of MG induced by the progression of the fatiguing task.

## Author contributions

Conceived and designed the experiments: RA and RE. Performed experiment: TF, MK, and MN. Analyzed data: RA and TF. Drafted manuscript and prepared tables and figures: RA. All authors interpreted results of research, edited, critically revised, and approved the final version of manuscript, and have agreed to be accountable for all aspects of the work related to its accuracy and integrity.

### Conflict of interest statement

The authors declare that the research was conducted in the absence of any commercial or financial relationships that could be construed as a potential conflict of interest.
